# Predictors of Disengagement and Loss to Follow-Up of Intravitreal Injection for Neovascular Age-Related Macular Degeneration in a Real-World Clinical Setting: Post Hoc Analysis of the Multicenter Survey from the Japanese Clinical Retinal Study (J-CREST) Group

**DOI:** 10.3390/jcm14061803

**Published:** 2025-03-07

**Authors:** Masaya Imazeki, Masaru Takeuchi, Tsutomu Yasukawa, Hiroto Terasaki, Yuki Yamamoto, Tatsuya Jujo, Makiko Wakuta, Hisashi Matsubara, Yoshinori Mitamura, Aki Kato, Mineo Kondo, Kazuhiro Kimura, Hitoshi Takagi, Fumi Gomi, Taiji Sakamoto

**Affiliations:** 1 Department of Ophthalmology, National Defense Medical College, 3-2 Namiki, Tokorozawa 359-8513, Japan; stage271828@gmail.com; 2 Department of Ophthalmology and Visual Science, Nagoya City University Graduate School of Medical Sciences, 1 Kawasumi, Mizuho-cho, Mizuho-ku, Nagoya 467-8601, Japan; yasukawa@med.nagoya-cu.ac.jp (T.Y.); pink299pu2pu2@gmail.com (A.K.); 3 Department of Ophthalmology, Kagoshima University Graduate School of Medical and Dental Sciences, Sakuragaoka 8-35-1, Kagoshima 890-8544, Japan; teracchi@m2.kufm.kagoshima-u.ac.jp (H.T.); tsakamot@m3.kufm.kagoshima-u.ac.jp (T.S.); 4 Department of Ophthalmology, Hyogo Medical University, 1-1, Mukogawacho, Nishinomiya 663-8501, Japan; yuki.kom.0923@gmail.com (Y.Y.); fgomi@hyo-med.ac.jp (F.G.); 5 Department of Ophthalmology, St. Marianna University School of Medicine, 2-16-1 Sugao, Miyamae-ku, Kawasaki 216-8511, Japan; t2jujo@marianna-u.ac.jp; 6 Department of Ophthalmology, Yamaguchi University Graduate School of Medicine, 1-1-1, Minami-Kogushi, Ube 755-8505, Japan; mwakut@yamaguchi-u.ac.jp (M.W.); k.kimura@yamaguchi-u.ac.jp (K.K.); 7 Department of Ophthalmology, Mie University Graduate School of Medicine, 2-174 Edobashi, Tsu 514-8507, Japan; hmatsu@med.mie-u.ac.jp (H.M.); mineo@med.mie-u.ac.jp (M.K.); 8 Department of Ophthalmology, Institute of Biomedical Sciences, Tokushima University Graduate School of Medicine, 3-18-15, Kuramoto-cho, Tokushima 770-8503, Japan; ymita@tokushima-u.ac.jp; 9 Kawasaki-Tama Eye Clinic, 2428, Noborito, Kawasaki 214-0014, Japan; htakagi@kt-eye.com

**Keywords:** anti-VEGF, AMD, PCV, RAP, neovascular AMD, treatment discontinuation, withdrawal

## Abstract

**Background/Objectives**: In a recent study, we investigated anti-VEGF treatment strategies for three subtypes of neovascular age-related macular degeneration (nAMD)—typical AMD (tAMD), polypoidal choroidal vasculopathy (PCV), and retinal angiomatous proliferation (RAP)—among a large cohort of Japanese patients. To further explore these findings, we conducted a post hoc analysis of this cohort to identify factors associated with the discontinuation of anti-VEGF therapy for nAMD in a real-world clinical setting. **Methods**: We collected medical records of patients newly diagnosed with nAMD who initiated intravitreal anti-VEGF antibody injection therapy. Patients were divided into two groups: those who continued anti-VEGF therapy for one year and those who discontinued treatment. Baseline best-corrected visual acuity, optical coherence tomography (OCT) findings, injection regimen, and the type of anti-VEGF antibody drug used were analyzed using univariate and multivariate analyses. **Results**: A total of 667 treatment-naïve nAMD patients initiated anti-VEGF agents and followed the therapy for 1 year. The one-year dropout rate in this study was 13%. Logistic regression analysis revealed that poor initial visual acuity and a PRN treatment regimen were significantly associated with higher odds of dropout. Age, gender, systemic factors, and the choice of intravitreal injection did not show any significant differences. **Conclusions**: Poor initial visual acuity and PRN treatment regimens may increase the risk of treatment dropout and should be carefully monitored.

## 1. Introduction

Age-related macular degeneration (AMD) is a chronic degenerative ocular disease with pathological changes in the macular region, and is a significant cause of vision loss leading to irreversible blindness in developed countries [1]. AMD is categorized into two subtypes: atrophy AMD and neovascular AMD (nAMD). Atrophy AMD is a chronic degenerative disease that typically results in varying degrees of visual impairment and can progress to profound vision loss [2]. nAMD is the presence of blood vessels that have disruptively invaded the retina. nAMD can be classified into three subtypes: typical AMD (tAMD), polypoidal choroidal vasculopathy (PCV), and retinal angiomatous proliferation (RAP). tAMD can be histopathologically classified into type 1 and type 2 macular neovascularization (MNV). Type 1 MNV is characterized by the presence of neovascularization beneath the retinal pigment epithelium (RPE). In early-phase fluorescein angiography (FA), it typically presents as a poorly defined hyperfluorescent lesion that gradually becomes more diffuse [3]. Type 2 MNV is characterized by neovascularization that extends subretinally beyond the RPE. In early-phase FA, it typically presents as a well-defined hyperfluorescent lesion, followed by marked leakage [3]. PCV is defined by polypoidal lesions at the periphery of type 1 MNV [4], and optical coherence tomography (OCT) demonstrates a steep elevation of the RPE. Indocyanine green angiography demonstrates well-circumscribed, round, nodular hyperfluorescent lesions at the periphery of type 1 MNV [5]. nAMD with type 3 MNV can also be referred to as RAP. Patients with RAP typically exhibit neovascularization within the retina, with subsequent anastomoses to retinal and choroidal vessels [6]. The first-line treatment for nAMD is intravitreal injection of anti-VEGF agents [7,8]. In representative clinical trials for nAMD, regardless of nAMD subtype, all tested drugs demonstrated improved visual acuity [9,10,11,12,13].

Recently, we performed a retrospective cohort study to examine the clinical features, treatment patterns, and visual outcomes in three subtypes of nAMD among a large cohort of Japanese patients in a real-world setting [14]. It was demonstrated that treatment regimens of aflibercept (2.0 mg) and ranibizumab (0.5 mg) were similar across the three subtypes. Patients received approximately five injections in the first year, irrespective of the anti-VEGF agent used. This was significantly less frequent in the pro re nata (PRN) group compared to the treat-and-extend (TAE) group. Aflibercept (2.0 mg) was used more frequently than ranibizumab and was administered to 70% of the overall nAMD patients. Visual acuity (VA) improved after 1 year of anti-VEGF therapy in all three subtypes; however, the improvement was not significant in the RAP subtype. Although nAMD patients who discontinued anti-VEGF treatments during the first year were excluded in this study, a major challenge in real-world clinical practice is patient non-adherence to hospital visits and intravitreal injections. Therefore, we conducted a post hoc analysis of nAMD patients who discontinued or were lost to follow-up within 1 year of anti-VEGF therapy in our previous study to identify the factors associated with treatment discontinuation and loss to follow-up.

## 2. Materials and Methods

### 2.1. Study Design and Participants

This multicenter retrospective cohort study was carried out at nine tertiary referral centers affiliated with the Japanese Clinical Retinal Study (J-CREST) Group. The study was conducted in accordance with the principles of the Declaration of Helsinki and was approved by the ethics committees of the National Defense Medical College (IRB number: 4353), Nagoya City University, Kagoshima University, Hyogo Medical University, St. Marianna University School of Medicine, Yamaguchi University, Mie University, Tokushima University, and Kawasaki Tama Eye Clinic. As this was a retrospective study, the requirement for written informed consent was waived by the ethics committees. Nevertheless, all study participants were provided with information regarding the study objectives and procedures.

In this multicenter retrospective cohort study, we consecutively enrolled patients newly diagnosed with nAMD and initiated treatment with ranibizumab (0.5 mg) or aflibercept (2.0 mg) between January 2014 and June 2019. We retrospectively collected the medical records of 706 patients newly diagnosed with nAMD at nine centers. Six patients were excluded due to a transfer to another institution, and thirty-three patients were excluded due to a lack of medical records. Due to the retrospective and post hoc study, the definition of treatment discontinuation was left to the discretion of the attending physician, and a uniform discontinuation criterion was not established. The 580 patients who were able to continue anti-VEGF treatment for 1 year were divided into the continuation group, and the 87 patients who stopped treatment were divided into the discontinuation group for analysis. In the analysis of the treatment discontinuation group, data from all 87 patients were used for the baseline analysis. For visual acuity and the number of clinic visits after 1 year, data from 49 patients who continued clinic visits for 1 year were analyzed.

### 2.2. Data Collection

The participating centers collected the following baseline and 1-year follow-up data from medical records: demographics; best-corrected visual acuity (BCVA) measured using Landolt C charts; spectral domain optical coherence tomography (SD-OCT) findings including intraretinal fluid (IRF), subretinal hyperreflective material (SHRM), subretinal fluid (SRF), subretinal hemorrhage (SRH), serous pigment epithelial detachment (PED), central retinal thickness (CRT), and central choroidal thickness (CCT); fellow eye status; details of anti-VEGF treatment (agent, regimen); the number of intravitreal injections; and the number of hospital visits. Additionally, systemic data including smoking history, diabetes, hypertension, cardiovascular disease, cerebrovascular disease, and anticoagulant use were obtained from clinician-completed questionnaires during routine patient visits and verified against medical records. For patients with bilateral nAMD, the study eye was defined as the eye in which symptoms first appeared. For patients with visual acuity of counting fingers, hand motion, light perception, or no light perception, logMAR values of 3, 4, 5, and 6 were assigned, respectively. As the visual acuity data did not follow a normal distribution, the median logMAR VA was calculated as an additional analysis. This study is a post hoc analysis, and there is some overlap in the analyzed data with our previous publication.

### 2.3. Outcome Measures

The primary outcome measures were the proportion of patients who discontinued anti-VEGF intravitreal injection within 1 year, the proportion of nAMD subtypes in both the continuation and discontinuation groups, and the selection of intravitreal injection regimen and injection drug.

### 2.4. Statistical Analysis

Statistical analyses were performed using JMP Pro version 17 (Business Unit of SAS, Cary, NC, USA). The normality of the data was analyzed by the Shapiro–Wilk test. Nonparametric variables were analyzed by the Mann–Whitney U test. The chi-square test was used to compare categorical data between groups. Logistic regression analysis was used to calculate the odds ratios of treatment discontinuation factors between the two groups. A *p* value less than 0.05 was considered to be statistically significant.

## 3. Results

### 3.1. Baseline Characteristics of Patients with Two Groups

Of the 667 treatment-naïve nAMD patients, 580 continued anti-VEGF intravitreal injections for 1 year, and 87 discontinued treatment (discontinuation rate: 13%). Table 1 shows patient characteristics and baseline data for the overall population and each group. The mean age was 75.5 ± 9.1 years in all nAMD patients, 75.5 ± 8.6 years in the continuation group, and 75.3 ± 11.9 years in the discontinuation group. The male to female ratio was 455: 212 overall, and the proportions of them in each group were similar and did not differ significantly between groups. With regard to systemic factors, there was a significantly higher proportion of smokers in the continuation group compared to the discontinuation group. However, no significant differences were observed in the proportions of patients with a history of diabetes, hypertension, cardiovascular disease, cerebrovascular disease, or anticoagulant use between the groups. Table 2 shows the proportions of nAMD subtypes in the overall patient population, the continuation group, and the discontinuation group. Among the 667 patients, 359 were classified as tAMD, 266 as PCV, and 42 as RAP overall. No significant differences in the proportions of subtypes were observed between the groups.

### 3.2. Baseline Visual Acuity and Ocular Findings of Two Groups

Table 3 shows the baseline visual acuity of the affected and fellow eyes in the overall patient group and in each subgroup. The mean logMAR VA in the affected eye was 0.32 ± 0.47 in the continuation group and 0.44 ± 0.58 in the discontinuation group. The mean logMAR VA in the fellow eye was 0.28 ± 0.58 in the continuation group and 0.39 ± 0.74 in the discontinuation group. There was no significant difference in baseline visual acuity between the two groups. OCT findings were as follows: the mean CRT was 312 ± 144 µm in the continuation group, and the mean CRT was 368 ± 194 in the discontinuation group, with a significantly thicker CRT in the discontinuation group than in the continuation group. The mean CCT was 288 ± 130 µm in the continuation group, and the mean CCT was 285 ± 123 in the discontinuation group. Serous PED was more frequently found in the discontinuation group than in the continuation group. There were no significant differences in SRF, IRF, SHRM, and SRH in both groups.

### 3.3. Anti-VEGF Agents Used and Treatment Strategies

All patients were treated with either ranibizumab (0.5 mg) or aflibercept (2.0 mg). A total of 221 patients (33.1%) were treated with ranibizumab and 446 (66.9%) with aflibercept, and the ranibizumab-to-aflibercept ratio did not differ significantly in both groups. The intravitreal injection regimen in all nAMD patients was PRN in 342 patients (51.4%), TAE in 281 patients (42.2%), and bimonthly in 43 patients (6.5%); these ratios were similar in the continuation group, but the ratio of PRN was significantly higher in the discontinuation group (Table 4).

### 3.4. Visual Acuity After 1 Year

The mean logMAR VA in the affected eye after 1 year was 0.29 ± 0.44 in the continuation group and 0.60 ± 0.90 in the discontinuation group. The mean logMAR VA in the fellow eye after 1 year was 0.18 ± 0.54 in the continuation group and 0.24 ± 0.83 in the discontinuation group. The mean log MAR VA in the affected eye after 1 year was significantly worse in the discontinuation group (Table 5).

### 3.5. Mean Number of Injection and Hospital Visits for 1 Year

Table 5 shows the number of injections and hospital visits for both groups. We analyzed patients in the discontinuation group who discontinued treatment but continued to follow up for one year. The mean number of injections for 1 year was 5.6 ± 2.4 in the continuation group and 3.6 ± 1.9 in the discontinuation group. The mean number of hospital visits for one year was 13.0 ± 6.1 in the continuation group and 10.4 ± 2.7 in the discontinuation group.

The number of hospital visits and injections was significantly lower in the discontinuation group than in the continuation group.

### 3.6. Discontinuation Factors of Anti-VEGF Treatment

To assess the odds ratio for treatment discontinuation, the following variables were analyzed: age, sex, Charlson Comorbidity Index (CCI), baseline logMAR visual acuity (VA) in the affected and fellow eyes, subtype of nAMD (tAMD, PCV, or RAP), and anti-VEGF regimen (PRN, TAE, or bimonthly). Patients with poor baseline best-corrected visual acuity in the affected eye were significantly more likely to discontinue treatment. The odds ratio for PRN treatment was significantly higher compared to TAE and bimonthly treatment. However, there were no significant differences in age, sex, CCI, or subtype of nAMD (Table 6).

## 4. Discussion

This post hoc study indicates that poor baseline visual acuity, PRN injection protocol, thick CRT, and serous PED are risk factors for treatment discontinuation.

Westborg et al. identified several risk factors for treatment discontinuation, including a baseline BCVA of less than 60 letters on the ETDRS chart, the use of ranibizumab (compared to aflibercept), treated at a university hospital, and a CCI score of 1 or more. Age and sex were not significant risk factors for treatment discontinuation [15]. Bakri et al. found that longer treatment intervals (≥12 weeks), female, and older age were associated with an increased risk of discontinuation. The other hand, CCI and the choice of anti-VEGF agent were not significantly associated with risk of discontinuation [16]. Cho et al. reported a retrospective study at a single center comparing nAMD patients who continued intravitreal injections with those who discontinued intravitreal injections. Patients who discontinued treatment due to no expectation of visual gain or poor response had significantly worse BCVA and a higher prevalence of diabetes compared to those who continued treatment and those who maintained remission for more than a year. Logistic regression analysis revealed that PCV and fewer intravitreal injections were significant risk factors [17]. Gillies et al. reported that older patients and those with poorer baseline visual acuity had higher dropout rates [18].

Our findings align with previous research, demonstrating that patients with poorer baseline visual acuity were more likely to discontinue treatment. In contrast, age, sex, the type of anti-VEGF agent used, and CCI did not significantly impact treatment discontinuation.

In our study, 13% of patients discontinued treatment within one year. Compared with clinical trials investigating the safety and efficacy of anti-VEGF agents for nAMD, the treatment discontinuation rate in our study was higher than those reported in clinical trials. The 1-year treatment discontinuation rate in the MARINA study, which evaluated the efficacy and safety of ranibizumab, was 10% [9]. The 1-year treatment discontinuation rate in the VIEW1 and VIEW2 studies, which evaluated the efficacy and safety of aflibercept, was 8.6% [11]. In contrast to previous real-world studies, our study demonstrated a lower rate of treatment discontinuation. For example, Hujanen et al. found a 1-year discontinuation rate of 26% [19], Rasmussen et al. reported 26.2% (with 10.8% due to inactive disease) [20], Basilious et al. found 28.8% [21], and Westborg et al. reported a remarkably high rate of 50.6% [15]. While our discontinuation rate was higher than that observed in clinical trials, it was lower than that reported in real-world settings.

The mean change in logMAR VA after one year was −0.03 in the continuation group and +0.16 in the discontinuation group. In contrast, the MARINA study reported a mean improvement of logMAR VA −0.14 in the group treated with 0.5 mg ranibizumab every 4 weeks [9], and the VIEW1/VIEW2 studies reported a mean improvement of logMAR VA −0.18 in the groups treated with 2 mg aflibercept every 4 or 8 weeks [11]. The untreated group in the MARINA study showed a mean worsening of 0.2 logMAR units [9]. In the continuation group in our study, the mean change in logMAR VA was smaller than the mean change in logMAR VA in patients who continued treatment for one year in the MARINA and VIEW1/VIEW2 studies. In the discontinuation group in our study, the mean change in logMAR VA was almost equivalent to the mean change in logMAR VA in the untreated group in the MARINA study. It is considered that treatment in clinical practice is more likely to be undertreated compared to clinical trials.

In previous studies investigating the long-term visual outcomes of nAMD, Starr et al. reported that more than 50% of patients with nAMD maintained a BCVA of 20/40 or better after 10 years of treatment [22]. Similarly, Brynskov et al. reported that in nAMD patients with a baseline visual acuity of 57.9 ± 16.4 letters on the ETDRS chart, visual acuity was maintained at 52.9 ± 2.2 letters after 10 years of treatment [23]. On the other hand, there is a report on the visual outcomes of patients who discontinued anti-VEGF therapy. Kim et al. investigated the visual outcomes of patients who had initiated anti-VEGF therapy and then discontinued treatment. They reported that the mean logMAR VA worsened significantly from 1.02 ± 0.20 at the time of treatment discontinuation to 1.60 ± 0.56 after 24 months [24]. Nguyen et al. reported a high rate of MNV reactivation after treatment cessation in patients with MNV that had been inactive for more than 3 months. In their study, 41% of patients experienced MNV reactivation within one year of treatment cessation, increasing to 79% by five years. The median time to reactivation was 504 days, and the mean visual acuity loss associated with reactivation was 4.2 letters. Although patients who resumed treatment after reactivation gained a mean of 1.2 letters of visual acuity, they ultimately experienced a net loss of 3.3 letters compared to their visual acuity at the time of treatment cessation [25].

Several studies have reported on the visual outcomes of patients with nAMD whose disease had stabilized, allowing for treatment discontinuation. Scoles et al., in the CATT study, followed nAMD patients for 3 years after 2 years of treatment with ranibizumab or bevacizumab, during which the disease had stabilized. They reported that while the mean ETDRS chart score at treatment discontinuation was 80.8 ± 6.6 letters, it remained favorable at 79.0 ± 5.5 letters after 3 years [26]. Adrean et al. reported on the visual outcomes and recurrence rates over 2 years in nAMD patients who underwent treatment discontinuation according to the treat–extend–stop (TES) protocol. A total of 37.3% of patients met the criteria for treatment discontinuation and had their treatment stopped. The mean visual acuity at the time of treatment discontinuation was 20/50 (improved from a baseline of 20/70), but within 2 years, 26.2% of patients experienced a recurrence, and visual acuity decreased to an average of 20/60. However, in patients who resumed treatment, visual acuity recovered to the level at the completion of the TES protocol. nAMD is a chronic condition, and regular intravitreal injections of anti-VEGF agents can help maintain visual acuity [27]. Previous studies have suggested that treatment discontinuation may be possible under certain circumstances.

This study has several limitations. We analyzed the risk factors for treatment discontinuation based on baseline characteristics, treatment regimens, and drug selection. However, as the individual reasons for treatment discontinuation were not examined in detail, it cannot be definitively concluded that these factors directly cause treatment discontinuation. This study predominantly involved university hospitals (eight out of nine centers), which may not be representative of the accessibility and quality of care for nAMD patients across Japan. Due to the involvement of nine centers and multiple attending physicians, inter-practice variations, which this study may not have captured, could have contributed to patient treatment discontinuation. Additionally, patients who transferred to other facilities may have continued treatment, potentially contributing to the imbalanced cohort analysis.

According to the study by Gomi et al. that used an online questionnaire to ask nAMD patients about their reasons for discontinuing treatment, physician-directed discontinuation was the most common reason for discontinuation [28]. Vaze et al. reported that in a clinical study of patients with nAMD, 42.3% discontinued treatment within a 6-year follow-up period. The primary reason for treatment discontinuation was the physician’s judgment that treatment was no longer necessary or that the treatment was ineffective [29]. Multiple studies have reported various reasons for patient treatment discontinuation, including dissatisfaction with the therapeutic efficacy of intravitreal injections, fear or pain associated with the intravitreal injections, financial burden, difficulty of hospital visit due to comorbidities, and death [29,30,31,32,33,34,35].

Our study showed that poor baseline visual acuity, thick CRT, and the presence of serous PED induce a decreased willingness of ophthalmologists to initiate treatment compared to patients without these characteristics. Furthermore, we observed a potential for treatment and clinic visit discontinuation due to patients’ high expectations for the therapeutic effect of anti-VEGF intravitreal injections, which can lead to difficulty in perceiving the actual treatment benefits. While a PRN injection regimen may be chosen to reduce the physical and economic burden of frequent injections, our study found that a PRN injection regimen may lead to treatment discontinuation. The choice of anti-VEGF agent, in this study ranibizumab and aflibercept, was not significantly different between the continuation and discontinuation groups.

## 5. Conclusions

To preserve patients’ visual acuity, physicians have to address risk factors for treatment discontinuation. Our study suggests that poor initial visual acuity and PRN regimens may be risk factors for treatment discontinuation.

## Figures and Tables

**Table 1:** Background data of patients with two groups of nAMD: continuation group and discontinuation group.

	Total nAMD (n = 667)	Continuation (n = 580)	Discontinuation (n = 87)	*p* Value *
Age, yrs				
Mean (SD)	75.5 (9.1)	75.5 (8.6)	75.3 (11.9)	0.6376
[95% CI]	[74.8, 76.2]	[74.7, 76.1]	[72.8, 77.8]	
Gender, *no* (%)				
Male	455 (68.2)	395 (68.1)	60 (69.0)	0.9025
Female	212 (31.8)	185 (31.9)	27 (31.0)	
Systemic factors, *n* (%)				
Smoking history (n = 444)	247 (55.6)	225 (57.5)	22 (41.5)	0.0384
Diabetes (n = 579)	117 (20.2)	102 (20.5)	15 (18.5)	0.7665
Hypertension (n = 578)	318 (55.0)	281 (56.4)	37 (46.3)	0.0918
Cardiovascular disease (n = 575)	84 (14.6)	69 (13.9)	15 (19.2)	0.2275
Cerebrovascular disease (n = 583)	50 (8.5)	41 (8.1)	9 (11.5)	0.2853
Anticoagulant agents (n = 428)	72 (16.8)	61 (16.7)	11 (17.5)	0.8564

* Comparison between continuation and discontinuation groups by Mann–Whitney U test for continuous variables and by chi-square test for categorical variables.

**Table 2:** 3 Subtypes of nAMD: tAMD, PCV, and RAP.

		Total nAMD (n = 667)	Continuation (n = 580)	Discontinuation (n = 87)	*p* Value *
Subtype of nAMD n (%)	tAMD	359 (53.8)	312 (53.8)	47 (54.0)	0.5424
	PCV	266 (39.9)	229 (39.5)	37 (42.5)	
	RAP	42 (6.3)	39 (6.7)	3 (3.5)	

nAMD = neovascular age-related macular degeneration; tAMD = typical age-related macular degeneration; PCV = polypoidal choroidal vasculopathy; RAP = retinal angiomatous proliferation. * Comparison between continuation and discontinuation groups by chi-square test.

**Table 3:** Visual acuity and ocular findings at baseline in two groups.

	Continuation (n = 580)	Discontinuation (n = 87)	*p* Value *
logMAR VA in affected eye			0.1909
Mean (SD) [95% CI]	0.32 (0.47) [0.28, 0.36]	0.44 (0.58) [0.32, 0.56]	
Median (range)	0.22 (−0.18–3)	0.22 (−0.18–3)	
logMAR VA in fellow eye			0.2682
Mean (SD)	0.28 (0.58) [0.23, 0.33]	0.39 (0.74) [0.23, 0.55]	
Median (range)	0 (−0.18–5)	0 (−0.18–5)	
Mean central retinal thickness in affected eye, µm (SD)	312 (144)	368 (194)	0.0465
[95% CI]	[300, 324]	[327, 409]	
Mean central choroidal thickness in affected eye, µm, (SD)	288 (130)	285 (123)	0.9404
[95% CI]	[277, 299]	[259, 311]	
SD-OCT findings, *n.* (%)			
IRF (n = 665)	171 (29.6)	30 (34.5)	0.3812
SHRM (n = 664)	232 (40.0)	37 (43.0)	0.6386
SRF (n = 667)	499 (86.0)	73 (83.9)	0.6215
SRH (n = 660)	198 (34.6)	29 (33.3)	0.9038
Serous PED (n = 667)	262 (45.2)	55 (63.2)	0.0018

SD-OCT = spectral domain optical coherence tomography; IRF = intraretinal fluid; SHRM = subretinal hyperreflective material; SRF = subretinal fluid; SRH = subretinal hemorrhage; PED = pigment epithelial detachment. * Comparison between continuation and discontinuation groups by Mann–Whitney U test for continuous variables and by chi-square test for categorical variables.

**Table 4:** Anti-VEGF agents used and treatment strategies.

	Total (n = 667)	Continuation (n = 580)	Discontinuation (n = 87)	*p* Value *
Anti-VEGF agents, *n* (%)				0.4644
Ranibizumab	221 (33.1)	189 (32.6)	32 (36.8)	
Aflibercept	446 (66.9)	391 (67.4)	55 (63.2)	
Injection protocol, *n* (%)				<0.001
PRN	342 (51.4)	269 (46.4)	73 (84.9)	
TAE	281 (42.2)	269 (46.4)	12 (14.0)	
Bimonthly	43 (6.5)	42 (7.2)	1 (1.2)	

PRN = pro re nata; TAE = treat and extend. * Comparison between continuation and discontinuation groups by chi-square test.

**Table 5:** Visual acuity of affected eye and fellow eye after 1 year and mean number of injection and hospital visits for 1 year.

	Continuation (n = 580)	Discontinuation (n = 49)	*p* Value *
logMAR VA in affected eye			
Mean (SD) [95% CI]	0.29 (0.44) [0.25, 0.33]	0.60 (0.90) [0.35, 0.85]	0.0193
Median (range)	0.15 (−0.18–4)	0.30 (−0.18–5)	
logMAR VA in fellow eye			
Mean (SD) [95% CI]	0.18 (0.54) [0.14, 0.22]	0.24 (0.83) [0.008, 0.47]	0.8462
Median (range)	0 (−0.18–4)	0 (−0.18–5)	
Mean number of injections for 1 year (SD)	5.6 (2.4)	3.6 (1.9)	<0.001
[95% CI]	[5.4, 5.8]	[3.1, 4.1]	
Mean hospital visits for 1 year (SD)	13.0 (6.1)	10.4 (2.7)	0.0016
[95% CI]	[12.5, 13.5]	[9.6, 11.2]	

* Comparison between continuation and discontinuation groups by Mann–Whitney U test.

**Table 6:** Logistic regression analysis of factors associated with anti-VEGF treatment discontinuation.

	Odds Ratio	*p* Value	95% CI of Odds Ratio	
			Lower limit	Upper limit
Age	1.00	0.87678	0.96	1.04
Gender (male/female)	0.98	0.94867	0.58	1.66
CCI	0.94	0.68773	0.67	1.30
logMAR VA in affected eye	1.57	0.04944	1.01	2.45
logMAR VA in fellow eye	1.28	0.16369	0.91	1.82
Subtype of nAMD PCV vs. tAMD	1.11	0.6811	0.68	1.82
Subtype of nAMD PCV vs. RAP	3.19	0.0758	0.89	11.47
Subtype of nAMD RAP vs. tAMD	0.35	0.1004	0.10	1.23
Anti-VEGF regimen PRN vs. TAE	6.16	<0.0001	3.25	11.68
Anti-VEGF regimen PRN vs. bimonthly	11.54	0.0169	1.55	85.89
Anti-VEGF regimen bimonthly vs. TAE	0.53	0.5532	0.07	4.25

CCI = Charlson Comorbidity Index; nAMD = neovascular age-related macular degeneration; tAMD = typical age-related macular degeneration; PCV = polypoidal choroidal vasculopathy; RAP = retinal angiomatous proliferation; PRN = pro re nata; TAE = treat and extend. Odds ratio for risk of discontinuation was calculated using logistic regression analysis with age, gender, Charlson Comorbidity Index score, logMAR VA in affected eye and fellow eye, subtype of nAMD, and injection regimen.

## Data Availability

Researchers can contact Masaru Takeuchi, MD, PhD, (masatake@ndmc.ac.jp) for details of the protocol and results.
